# Ongoing disruption of RSV epidemiology in children in Switzerland

**DOI:** 10.1016/j.lanepe.2024.101050

**Published:** 2024-09-02

**Authors:** Patrick M. Meyer Sauteur, Margherita Plebani, Johannes Trück, Noémie Wagner, Philipp K.A. Agyeman, RSV EpiCH investigators

**Affiliations:** a Division of Infectious Diseases and Hospital Epidemiology, Children's Research Center, University Children's Hospital Zurich, University of Zurich, Zurich, Switzerland; b Pediatric Infectious Diseases and Vaccinology Unit, Department Women-Mother-Child, Lausanne University Hospital, Lausanne, Switzerland; c Divisions of Allergy and Immunology, Children's Research Center, University Children's Hospital Zurich, University of Zurich, Zurich, Switzerland; d Pediatric Infectious Diseases Unit, Division of General Pediatrics, Department of Woman, Child, and Adolescent Medicine, Geneva University Hospitals, Geneva, Switzerland; e Division of Pediatric Infectious Disease, Department of Pediatrics, Inselspital, Bern University Hospital, University of Bern, Switzerland

The RSV EpiCH project aims to provide representative nationwide surveillance on respiratory syncytial virus (RSV) infections in children in Switzerland.^1^ Prior to the COVID-19 pandemic, Switzerland had observed stable biennial cycles of alternating strong and weak RSV winter epidemics in children, as reported for some countries at northern latitudes.^2^^,^^3^ The almost complete suppression of RSV circulation in children in the early phase of the COVID-19 pandemic was followed by an exceptional out-of-season-activity in summer 2021 during lifting of non-pharmaceutical interventions (NPIs).^1^^,^^4^ Here, we present findings from the ongoing surveillance of RSV in children in Switzerland since January 2021.

The RSV EpiCH project prospectively collects weekly data on the detection of RSV in children from 21 of 29 paediatric acute care hospitals in Switzerland.^1^ These hospitals represent >90% of available paediatric beds in Switzerland. All sites reported the number of children with detection of RSV. We included all detections including results of rapid tests, and non-PCR-based methods. There was no standardized testing strategy imposed on sites participating in RSV EpiCH. Consequently, some sites tested in- and outpatients, some sites used targeted testing (only testing children with bronchiolitis) and some untargeted testing (testing all children with respiratory disease). These study characteristics and testing strategies of participating sites are listed in Table S1. Aggregated and anonymised data about RSV in children included the number of tests, detections, and detections per age group (infants, 1-year-old children, and ≥2-years-old children).^1^

The following three time periods were compared: January 2021–June 2022 (2021/2022 pandemic period, including winter seasons 2020/2021 and 2021/2022); July 2022–June 2023, first winter season after NPIs had been lifted (2022/2023 winter season); and July 2023–June 2024 (2023/2024 winter season). Seasonality was not specifically accounted for in the analysis of age distribution or the comparison of the differences between regions. During the study period Palivizumab was the only available drug for prevention of RSV infection in infants in Switzerland and was only recommended for infants with chronic lung disease or hemodynamically significant heart disease.^1^

From 4th January 2021 to 30th June 2024, RSV was detected in 15'073 children. The detections were distributed over the defined periods as follows: 5'160 (incidence rate 2.3 per 1000 children-years) in the 2021/2022 pandemic period, 5'242 (3.5 per 1000 children-years) in the 2022/2023 winter season, and 4'671 (3.1 per 1000 children-years) in the 2023/2024 winter season (Fig. 1a). Detections were reported from 8909 (59%) infants, 2866 (19%) from 1-year-old children, and 3298 (22%) from ≥2-years-old children. Age distribution of children with RSV infection varied significantly between the three periods (Pearson $\chi^{2}$-test p < 0.001, Table S2). The 2021/2022 pandemic period was characterized by continuous RSV detections in all participating hospitals during summer and autumn 2021, as well as in winter 2021/2022. However, we observed regional differences with regions in the central, eastern and south-eastern parts of Switzerland showing more similar evolution of the RSV counts than the western part (Fig. 1b and Fig. S1). The first two post pandemic winter seasons have shown a return to occurrence of RSV in cold months only and a harmonization of the time series of RSV detection counts between the different parts in Switzerland (Fig. S1). We have historical data for 2 sites and sufficiently granular data for the University Children's Hospital Bern only. Using a susceptible-infected-recovered-susceptible (SIRS) dynamic transmission model with waning immunity with the lock-down period as a time-varying input we can show that a return to the previously expected alternating cycle of strong and weak seasons would have been expected. However, we have observed an earlier start of the 2022/2023 winter season, which was exceptionally strong compared to historical data, and an unexpected sequence of two strong RSV seasons on top of each other (Fig. 1a and Fig. S2).

**Fig. 1:**
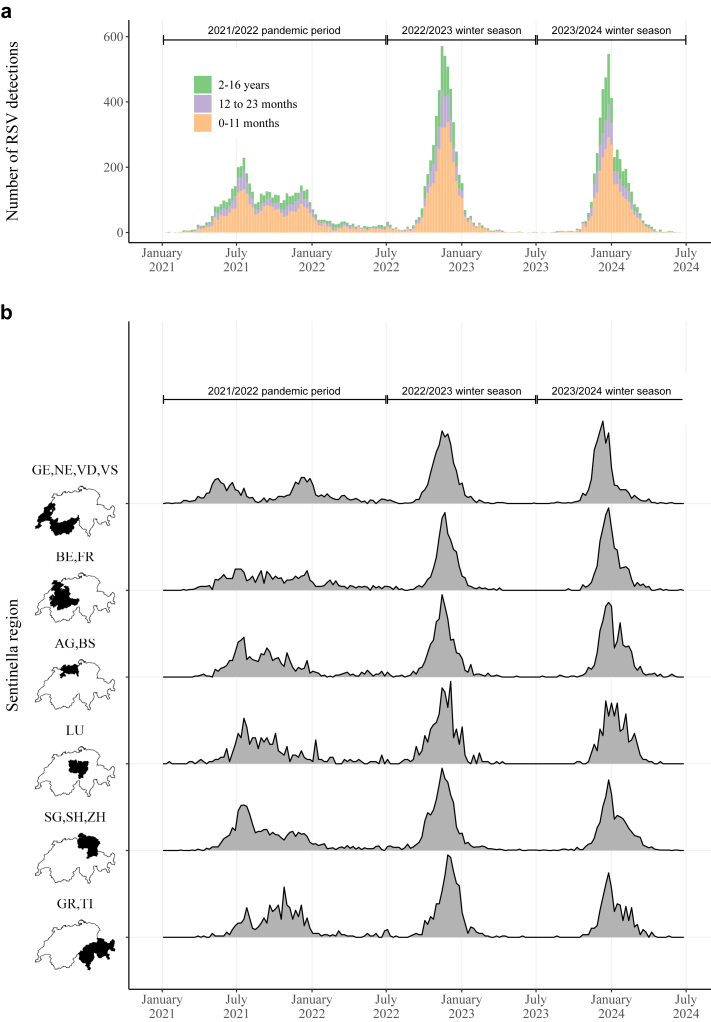
Weekly number of RSV detections in children in Switzerland January 2021–June 2024. a) Absolute numbers by age group. b) Scaled relative numbers for the 6 respiratory virus reporting regions in Switzerland. Scaling was done within each Sentinella region, ensuring that the maximum height of each curve corresponds to the maximal number of RSV detections over the whole study period in the respective region. GE, Geneva University Hospital; VD, Lausanne University Hospital; NE, Cantonal Hospital Neuchatel; VS, Valais Hospital and Hospital Riviera-Chablais; BE, University Hospital Bern and Children's Hospital Wildermeth, Biel; FR, Fribourg Hospital HFR; AG, Cantonal Hospital Aarau and Hospital Baden; BS, University Children's Hospital Basel; LU, Children's Hospital of Central Switzerland, Lucerne; SG, Children's Hospital of Eastern Switzerland; SH, Cantonal Hospital Muensterlingen; ZH, Cantonal Hospital Winterthur, University Children's Hospital Zurich and City Hospital Triemli, Zurich; GR, Cantonal Hospital of Graubuenden; TI, Institute of Pediatrics of Southern Switzerland (Bellinzona, Locarno, Lugano).

These data show an ongoing disruption of RSV epidemiology in children in Switzerland more than 1 1/2 years after NPIs against COVID-19 were lifted in Switzerland. However, the RSV epidemiology seems to shift back to a seasonal pattern as has been seen in other countries.^4^ Existing theories for the strong re-emergence of RSV after NPIs were lifted (e.g., waning immunity, decrease of transfer of RSV-specific maternal antibodies)^4^^,^^5^ are less likely to account for two consecutive strong seasons as observed in Switzerland. The observed fluctuations in the age distribution may indicate that there are ongoing changes in the epidemiology of RSV after the cessation of NPIs. While we have missing data for 2 regional hospitals from December 2021 until December 2022, we did not adjust our analyses for this. Despite this limitation, we assume that due to the extensive coverage of the network the data analysed here can be considered generalizable.

In conclusion, the RSV EpiCH project provides valuable insights into the dynamics of RSV activity in children in Switzerland, and will allow to assess the impact of the introduction of the monoclonal antibody Nirsevimab for preventing RSV in children in Switzerland.

## Contributors

PMMS, JT, and PKKA conceptualised the study. All authors collected and interpreted the data. PMMS, and PKKA wrote a first draft of the manuscript. All authors read and revised the manuscript and approved the final version.

## Data sharing statement

No individual participant data is collected in this study. Weekly aggregated number of RSV detections in children in participating hospitals are available to researchers on reasonable request. Please address inquiries with a methodologically sound proposal to philipp.agyeman@insel.ch.

## Declaration of interests

PMMS, JT and PKAA have participated in advisory board meetings for Nirsevimab organised by Sanofi. JT has received speaker fees from Sanofi and is member of a data safety monitoring board for mRNA-based RSV vaccines by Moderna.
