# Supplementary material for: Ongoing disruption of RSV epidemiology in children in Switzerland
Source: Lancet Reg Health Eur. 2024 Sep 2;45:101050. doi: 10.1016/j.lanepe.2024.101050 (PMC11403506; doi:10.1016/j.lanepe.2024.101050)
Supplement: Supplementary Figures and Tables [file mmc1.pdf]

Supplementary Appendix

Ongoing disruption of RSV epidemiology in children in Switzerland

Patrick M. Meyer Sauteur, Margherita Plebani, Johannes Trück, Noémie Wagner, Philipp K. A. Agyeman on behalf of the RSV EpiCH investigators

Table of content

RSV EpiCH investigators 2
Supplementary table 1 3
Supplementary table 2 4
Supplementary figure 1 5
Supplementary figure 2 6
Role of funding sources 7
Acknowledgements 7

RSV EpiCH investigators

Patrick M. Meyer Sauteur[a], Christoph Aebi[b], Florence Barbey[c], Christoph Berger[a], Julia A. Bielicki[d], Michael Buettcher[e,f,g], Gaud Catho[h], Antony Croxatto[i], Beate Deubzer-Raunhardt[k], Alexis Dumoulin[h], Mathias U. Gebauer[l], Mirjam Grimm[m], Ulrich Heininger[d], Christian R. Kahlert[n], Eva Kellner[o], Lisa Kottanattu[p], Jasr Kawkby[o], Guido F. Laube[q], Anita Niederer-Loher[n], Margherita Plebani[r], Klara M. Posfay-Barbe[s], Nicole Ritz[e], Fabian Spigariol[t], Johannes Trück[u], Noémie Wagner[s], Petra Zimmermann[v], Franziska Zucol[w], Philipp K. A. Agyeman[b]

[a] Divisions of Infectious Diseases and Hospital Epidemiology, University Children's Hospital Zurich, Zurich, Switzerland

[b] Department of Pediatrics, Inselspital, Bern University Hospital, University of Bern, Switzerland

[c] Pediatric Infectious Diseases, Children's Hospital Aarau, Switzerland

[d] Pediatric Infectious Diseases, University of Basel Children's Hospital, Basel, Switzerland

[e] Paediatric Infectious Diseases, Department of Paediatrics, Children's Hospital of Central Switzerland, Lucerne, Switzerland

[f] Faculty of Health Sciences and Medicine, University of Lucerne, Lucerne, Switzerland

[g] Paediatric Pharmacology and Pharmacometrics Research Center at University Children's Hospital Basel, Basel, Switzerland

[h] Infectious Diseases Division, Central Institute, Valais Hospital, Sion, Switzerland

[i] Microbiologie, ADMED Analyses et Diagnostiques Médicaux, La Chaux-de-Fonds, Switzerland

[k] Kantonsspital Graubunden, Chur, Switzerland

[l] Kinderklinik Wildermeth am Spitalzentrum Biel, Biel-Bienne, Switzerland

[m] Kinderklinik Kantonsspital Müsterlingen, Switzerland

[n] Children's Hospital of Eastern Switzerland, St Gallen, Switzerland

[o] Klinik für Kinder und Jugendliche, Stadtspital Zürich, Switzerland

[p] Institute of Pediatrics of Southern Switzerland, EOC, Bellizona, Switzerland

[q] Department of Pediatrics, Hospital Baden, Baden, Switzerland

[r] Pediatric Infectious Diseases and Vaccinology Unit, Department Women-Mother-Child, Lausanne University Hospital, Lausanne, Switzerland

[s] Unité d'infectiologie pédiatrique, Service de pédiatrie générale, Département de la femme, de l'enfant et de l'adolescent, HUG, Genève, Switzerland

[t] Pédiatrie, Réseau hospitalier neuchâtelois, Switzerland

[u] Division of Allergy and Immunology, University Children's Hospital Zurich, Zurich Switzerland

[v] Faculty of Science and Medicine, University of Fribourg, Switzerland and Department of Paediatrics, Fribourg Hospital HFR, Fribourg, Switzerland

[w] Paediatric Infectious Diseases, Department of Paediatrics, Cantonal Hospital Winterthur, Switzerland

Supplementary table 1

Hospital	Hospital type	Testing strategy	Age group tested	Test modality	Sentinella region
Geneva University Hospital	Tertiary care hospital	Inpatient only (targeted)	0-16 years	PCR, Antigen	1
Lausanne University Hospital	Tertiary care hospital	Inpatient only (targeted)	0-18 years	PCR	1
Reseau hospitalier neuchatelois	Regional hospital	Inpatient only (targeted)	0-16 years	PCR	1
Hopital du Valais and Hopital Riviera-Chablais	Regional hospital and local hospital	In- and outpatient (targeted)	0-16 years	PCR	1
University Hospital Bern	Tertiary care hospital	Inpatient only (untargeted)	0-16 years	PCR, Antigen	2
Kinderklinik Wildermeth am Spitalzentrum Biel	Regional hospital	Inpatient only (untargeted)	0-16 years	PCR	2
Fribourg Hospital HFR	Regional hospital	Inpatient only (targeted)	0-16 years	PCR	2
Cantonal Hospital Aarau	Regional hospital	April–September: in- and outpatient (targeted). October–March: inpatient only (untargeted)	0-18 years	PCR, Antigen	3
Cantonal Hospital of Baden	Regional hospital	In- and outpatient (targeted)	0-16 years	PCR, Antigen	3
University Children's Hospital Basel	Tertiary care hospital	In- and outpatient (untargeted)	0-16 years	PCR	3
Children's Hospital of Central Switzerland, Lucerne	Tertiary care hospital	Inpatient only (targeted and untargeted)	0-16 years	PCR, Antigen	4
Cantonal Hospital Muensterlingen	Regional hospital	Inpatient only (untargeted)	0-16 years	PCR	5
Children's Hospital of Eastern Switzerland	Tertiary care hospital	Inpatient only (targeted)	0-16 years	PCR	5
Cantonal hospital of Winterthur	Regional hospital	In- and outpatient (targeted)	0-16 years	PCR	5
University Children's Hospital Zurich	Tertiary care hospital	Inpatient only (untargeted)	0-16 years	PCR	5
Triemli Hospital Zurich	Regional hospital	In- and outpatient (targeted)	0-16 years	PCR	5
Cantonal Hospital of Graubuenden	Regional hospital	Inpatient only (untargeted)	0-16 years	PCR	6
Istituto Pediatrico della Svizzera Italiana (Locarno, Bellinzona, Lugano, Mendrisio), Ente Ospedaliero Cantonale	Regional hospitals	Inpatient only, occasionally outpatient (targeted)	0-16 years	PCR, Antigen	6

Characteristics of hospitals participating in RSVEpiCH and their strategy for RSV testing. PCR=polymerase chain reaction; Antigen=RSV rapid antigen test or direct immunofluorescence.

Supplementary table 2

Period	0-11 months	12-23 months	2-18 years
2021/2022 pandemic period	3019 (58.5%)	1085 (21%)	1056 (20.5%)
2022/2023 winter season	3223 (61.5%)	913 (17.4%)	1106 (21.1%)
2023/2024 winter season	2667 (57.1%)	868 (18.6%)	1136 (24.3%)

Age distribution of children with RSV in Switzerland, stratified by season. Age distribution varied significantly between the three time periods ($p < 0.001$ from Pearson χ^2-test for all three time periods combined, $p < 0.001$ for the comparison 2021/2022 pandemic period vs. 2022/2023 winter season, $p < 0.001$ for the comparison 2021/2022 pandemic period vs 2023/2024 winter season, and $p < 0.001$ for the comparsion 2022/2023 winter season vs 2023/2024 winter season).

Supplementary figure 1

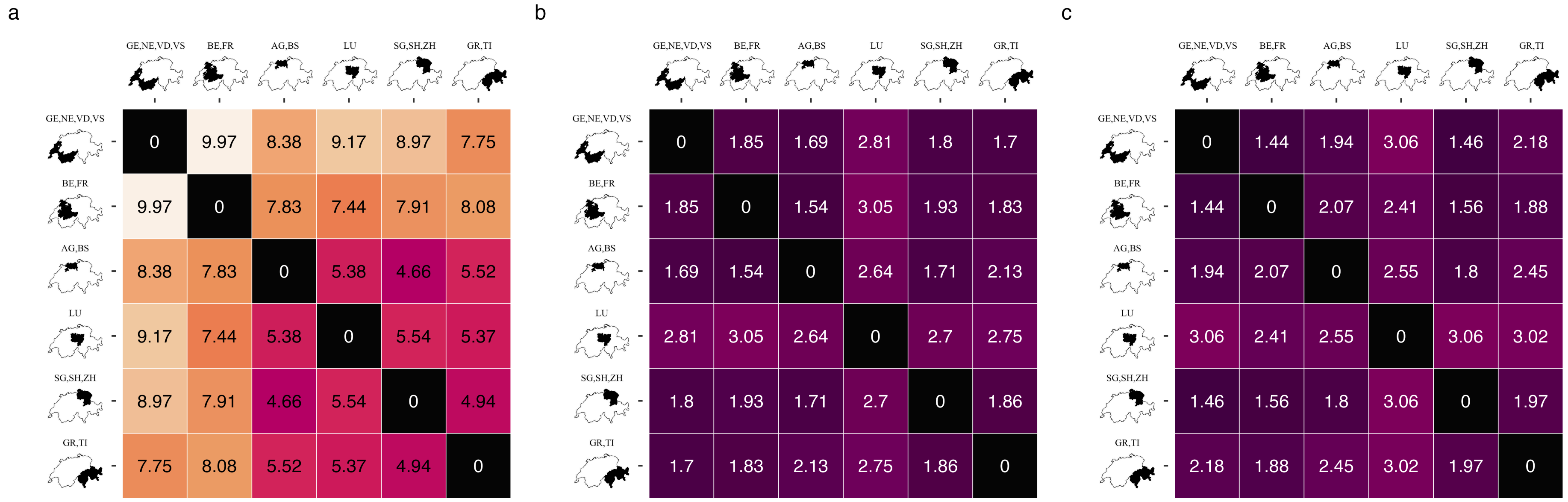

Heatmap of the pairwise similarity of the scaled time series of RSV detection in the 6 respiratory virus reporting regions in Switzerland for (a) the 2021/2022 pandemic period, (b) the 2022/2023 winter season, and (c) the 2023/2024 winter season. Similarity was measured by calculating the “distance” between time series using Dynamic Time Warping algorithm. Distance scores for the comparison of two time series are shown in the respective cell. Higher distance scores (light colours) indicate time series are more dissimilar while lower distance scores (dark colours) indicate time series are more similar. Panel (a) demonstrates that during the 2021/2022 pandemic period scaled time series of RSV detection in the northern, eastern and south-eastern regions of Switzerland clustered closer together compared to the western parts of Switzerland. Panels (b) and (c), in contrast, show RSV detection time series of all 6 regions of Switzerland were more similar during the 2022/2023 and 2023/2024 winter seasons compared to the 2021/2022 pandemic period.

Supplementary figure 2

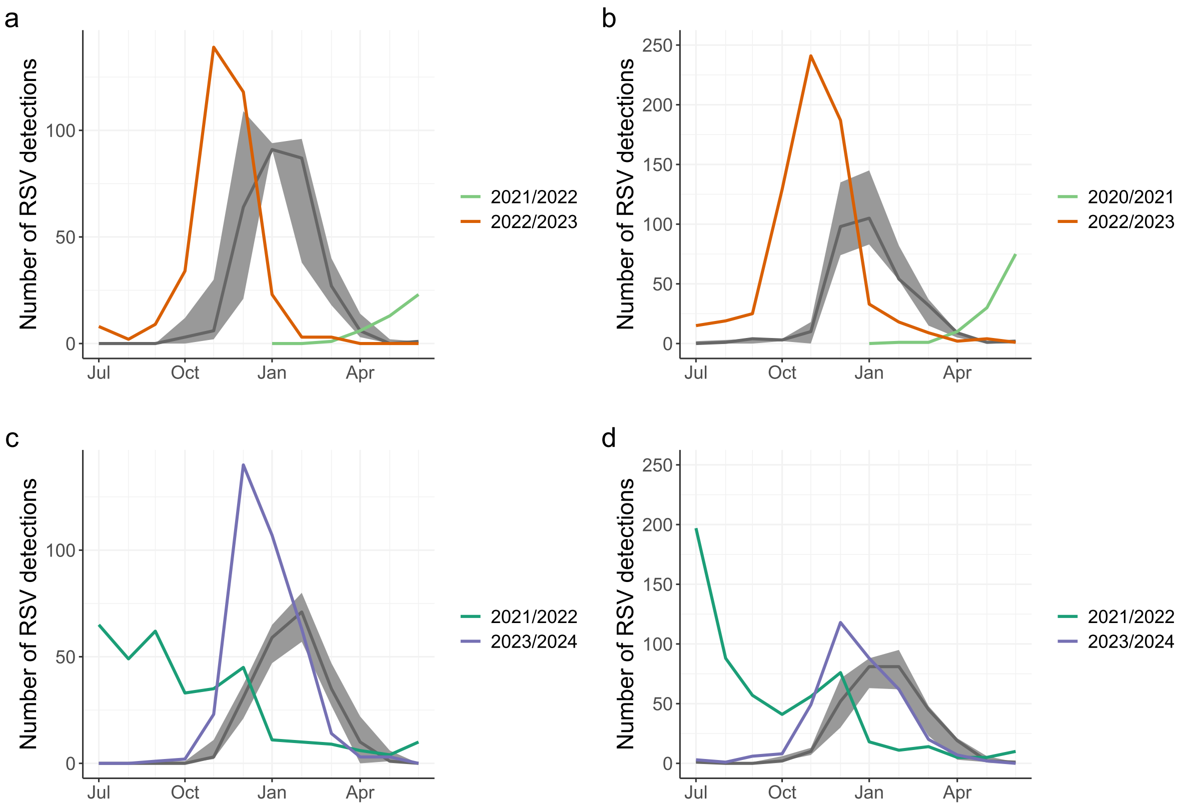

Monthly number of RSV detections in children in the University Hospitals Bern and Zurich by season strength, July 2014 - June 2024.
Data are shown for winter seasons starting in even-numbered years (strong seasons) for Bern (a) and Zurich (b) and winter seasons starting in odd-numbered years (weak seasons) for Bern (c) and Zurich (d) respectively. The light green line denotes the number of RSV detections from 4th January 2021 until 4th July 2021 (2021/2022 pandemic period), the dark green line from 5th July 2021 until 3rd July 2022 (2021/2022 pandemic period), the orange line the 2022/2023 winter season, and the violet line the 2023/2024 winter season. Historical data are shown in grey. The dark grey line indicates the median number, the light grey ribbon the minimum and maximum number of RSV detections by month for 2014/2015, 2016/2017, and 2018/2019 (strong seasons) and 2015/2016, 2017/2018, and 2019/2020 (weak seasons).

Role of funding sources

RSV EpiCH is not supported by external funding.

Acknowledgements

The authors would like to acknowledge the help of Valentin von Niederhäusern and Alix L. von Hammerstein with setting up the RSV EpiCH network and its database infrastructure. We also thank M. Hostettler, Fabiola Hadzic, and D. Bandeira for their help with collecting the data.
